# Wash durability and optimal drying regimen of four brands of long-lasting insecticide-treated nets after repeated washing under tropical conditions

**DOI:** 10.1186/1475-2875-9-248

**Published:** 2010-08-30

**Authors:** Francis K Atieli, Stephen O Munga, Ayub V Ofulla, John M Vulule

**Affiliations:** 1 Kenya Medical Research Institute, Centre for Global Health Research, Kisumu, Kenya; 2 Department of Biomedical Sciences, Maseno University, Kenya

## Abstract

**Background:**

The current study was undertaken to determine the optimal wash-drying regimen and the effects of different washing procedures on the efficacy, and durability of four brands of newly introduced long-lasting insecticide-treated nets (LLINs) under tropical conditions.

**Methods:**

In the current study, the following four LLINs were tested: Olyset^®^, PermaNet ^®^2.0, BASF^® ^and TNT^®^. Nets were divided into three sets; one set was washed by hand rubbing and air-dried either hanging or spread on the ground in direct sunlight or hanging or spread on the ground under the shade. A second set was washed using the WHO protocol (machine) and the third set was washed by beating the nets on rocks. The biological activities of the nets were assessed by a three-minute bioassay cone test and the residual insecticide contents were determined using high performance liquid chromatography (HPLC) procedure.

**Results:**

Nets that were dried hanging under the shade retained more insecticide, 62.5% and recorded higher mortality compared to nets which were dried lying on the ground in direct sunlight 58.8%, nets dried under the shade spread on the ground 56.3%, and 57.8% for nets dried hanging in direct sunlight. It was also observed that nets washed by the standard WHO protocol, retained more insecticide and were more effective in killing mosquitoes compared to nets washed by local methods of hand rubbing and beating on rocks. There were significant differences between drying regimens (p < 0.0001) and between washing procedures (p < 0.001) respectively. However, the effect of net type was statistically insignificant. The statistical differences on individual nets were also compared, for PermaNet^® ^and TNT there were no significant differences observed between the four drying regimens (*p *= 0.7944 and 0.4703) respectively). For BASF and Olyset, the differences were significant (p < 0.001 and p > 0.0001).

**Conclusion:**

The results of this study suggest that washing and drying regimen influence the insecticidal activity of LLINs. The standard WHOPES washing protocol underestimates the amount of insecticide washed from LLINs compared to the abrasive washing procedures that are used in the field. This suggests that there is need to educate net users to adopt a more gentle washing procedure while handling LLINs. The education should accompany net distribution campaigns.

## Background

Long-lasting insecticide-treated nets (LLINs) are currently preferred to conventionally insecticide-treated nets (ITNs) for use in malaria control programmes [1-4]. Although LLINs offer an alternative solution to regular net re-treatment, their actual wash durability under field conditions is not known. For example the frequency of washing, the washing methods, and drying regimens that are used in the field are some of the factors which are likely to affect their efficacy and durability. The standard WHOPES recommended washing procedure is not applicable under field conditions and might be underestimating the abrasive washing practices that are used in the field and, therefore, overestimating the biological durability of LLINs in many countries [5,6]. The manufacturers of LLINs, suggest that these kind of products do not require any further re-treatment throughout their lifetime, because they are treated with special binders which are wash durable [7]. Several studies undertaken under specific environmental conditions have since confirmed that LLINs offer longer time protection than conventional ITNs, [8-10]. The challenge is now to understand how these products will perform under various climatic conditions in the tropics where they are expected to be used.

Past studies have demonstrated different wash resistance of different brands of LLINs from one region to the other and even between laboratories [11,12]. Due to these differences, WHOPES has provided guidelines on standardized procedures for washing and drying of LLINs for the purposes of comparison of similar products between different laboratories [13]. However, it may not be possible to standardize washing methods in every region or country due to local economics and general practices. It is, therefore, necessary to undertake studies like this, so as to understand and document how different washing and drying regimens can affect the performance of these new innovations and give guidelines on the best washing and drying procedures.

LLINs have been promoted for use in reducing human-vector contact for a long time. However, there is limited information on the effect of different drying regimens and washing methods and how they affect the efficacy of current brands of LLINs, especially under tropical conditions. A recent study in Iran showed that the effect of exposing PermaNet for a short period to direct sunlight during drying was much smaller, and that drying nets for long hours in the sun is not harmful [14]. It has also been demonstrated that direct sunlight is harmful to pyrethroid-based insecticide because *uv *rays break down pyrethrin molecules thereby rendering the insecticide ineffective [15]. However, the role played by sunlight in enhancing or rendering the pytherins ineffective still remains, controversial. Two studies conducted in India, [16] and [17], showed that exposing Olyset to sunlight for a few hours enhances efficacy. On the 4^th ^update report on LLINs, WHO after reviewing several studies on efficacy of Olyset, recommended placing the nets in polythene bags and exposing them to sunlight for a few hours to enhance heat assisted insecticide migration to the surface at the same time preventing the effect of UV light after washing [1]. It has also been documented that heat accelerates the rate of migration of permethrin molecules in the fibers, thereby increasing the bioavailability of insecticide on the net surface, especially if they are polyethylene based. In one laboratory study [18] Olyset and PermaNet were equally exposed to a controlled temperature of 60°C for 4 hrs after repeated washing, only Olyset, regenerated. Heat and not sunlight appears to be the main contributing factor in the regeneration of polyethylene based nets. In the current study, the washing durability and different drying regimens of four brands of LLINs under local conditions were evaluated. Two of the LLINs; BASF and TNT, have recently been approved by World Health Pesticide Evaluation Scheme (WHOPES) and there is no information about their performance under field conditions. The study also compared the traditional washing methods of hand rubbing and beating on rocks commonly used in local villages to the standard WHO recommended procedure that uses a standard washing machine and a specific soap

## Methods

### Nets used in the study

Four brands of LLINs were evaluated. The nets were randomly selected from a consignment supplied by different manufacturers for field distributions. A total of 20 net samples were used as follows, four from each treatment group:

(i) Olyset (Sumitomo Corp.) production licensed to A - Z Tanzania. These are polyethylene-based nets. Treatment is done at the factory, with a synthetic permethrin @ 1,000 mg/m²

(ii) PermaNet^® ^2.0 (Vestergaard-Fransden, Denmark). These are polyester based nets and treatment is applied topically at the factory with deltamethrin @ 55 mg/m²

(iii) Interceptor (BASF, Germany) also polyester based. Treatment is done at the factory with alphacypermethrin @ 200 mg/m².

(iv) NetProtect (TNT) (Intelligent Insect control Co. France). They are polyethylene based and treatment is accomplished at factory with deltamethrin @ 65 mg/m². The production of these nets is licensed to Siva Enterprises of India.

A conventionally treated net with deltamethrin @ 25 mg/m² was used as a control. Nets were tested for bioactivity before washing to determine the baseline efficacy and thereafter they were tested after every 5^th ^wash through wash 20.

### Washing of nets by hand

The nets were hand washed and dried outdoor at KEMRI Centre for Global health research in Kisian village, western Kenya. Four field assistants from the local community were hired to do the washing. Hand washing was done by immersing the netting in a measured volume of water using a measured local detergent Omo. The field assistants were randomly assigned to wash the four brands of nets by hand rubbing in a 10-litre water bowl. Nets were washed for 10 minutes in 2 litres of cold rain water mixed with 5 g of detergent. After washing each net was rinsed twice for 5 minutes in same amount of clean water. Each net was washed twice a week. Washing was done mid-morning between 9 to 10 am.

### Net drying procedures

The four different drying regimens were tested only on nets that were washed by hands. It is important to note that this is the most common washing method used in the local villages. After washing, the nets were air-dried as follows: one net from each treatment group was air-dried in the sun by hanging, while the second net was air-dried in direct sunlight spread on the ground. The third net from each group was air-dried under the shade spread on the ground and fourth net from each treatment group was dried under the shade hanging on a line. The nets were left in position to dry for a fixed period of 4 hrs. Initially before adopting a standard drying time, nets were inspected hourly to ascertain their drying status. It was established that 4 hours was the adequate period in the study area for nets to dry completely, whether in direct sunlight or under the shade. This period was adopted as the optimal time nets needed to dry and was used throughout the study

### Washing of nets by machine

In this method, the net samples were washed using WHO protocol as follows: 1 gram of soap OMO powder was thoroughly dissolved in 500 ml of rain water in 1 L Erlenmeyer conical flask. A single sample of netting 30 by 30 cm was placed in the soap solution. The nets and soap solution were shaken for 10 min on an orbital shaker bath (C76 Water Bath Shaker; New Brunswick Scientific Co., Edison, NJ, USA) at 155 rotations per minute at room temperature. After washing with soap, the nets were rinsed twice by shaking for 10 min in 500 ml of rain water each. After the nets were rinsed a second time they were hung on a line indoors to dry for 4 hrs.

### Washing by beating on rocks

In this experiment, 5 grams of OMO detergent was thoroughly mixed with 2 litres of rain waster. Each net was individually immersed in soap solution in a 10-litre water bowl. Nets were then removed from the water bowl then beaten against a concrete slab several times for a total of 10 minutes. Between these beatings, the nets were momentarily soaked in same soap solution for a few seconds when they appeared to run out of dipping water. This was to simulate what happens in the local village. After washing with soap, each net was then rinsed twice for five minutes in same amount of clean water. Each net was washed twice a week. Washing was done mid-morning between 9 to 10 am. After washing and rinsing, nets were dried by hanging under the shade for 4 hours.

### Laboratory procedure

Four replicate net samples in templates measuring 30 × 30 cm were cut from each net brand and tested. In total, 20 samples were tested. Each net sample was subjected to a baseline bioassay before washing was started. After the baseline bioassay, samples were washed according to either the standard WHO protocol or a cording to local methods. Thereafter, the samples were washed twice a week. Subsequent bioassays were carried out after every 5^th ^wash on each 2^nd ^day after the previous washing till the 20^th ^wash. Tested samples were stored until after the end of the study for chemical residue analysis. The same samples were used for bioassay and chemical residue analysis. Tested samples were taken as follows; one from the top and three from each of the four sides at random

### Bioassays procedures

Four WHO cones were attached to each net sample and a total of 10 mosquitoes were introduced into each cone. 2-5 day old, unfed female laboratory reared susceptible Kisumu strain of *Anopheles gambiae *mosquitoes was used. At least 40 mosquitoes were exposed on each net for 3 min and then transferred to holding paper cups and provided with 5% sugar solution soaked in absorbent cotton pad. Knockdown was recorded after 30 minutes and 60 minutes after exposure. Mortality was recorded 24 hrs after exposure. Mosquitoes were considered knocked down or dead if they could not fly and could not stand upright on either the side or the bottom of the paper cups. Untreated net was used as a control and was tested each day the bioassay was performed. Tests were done at temperatures of 25°C ± 2 and 80% ± 10 relative humidity.

### Residual insecticide quantification

After bioassay the second piece cut from each net were individually labelled with the name of treatment group and number of washes and stored in the dark for subsequent residual insecticide quantification by high performance liquid chromatography (HPLC), [19]. For ease of identification the nets were marked with indelible ink using permanent markers before washing was started. The insecticide content of each net was determined by cutting from each net piece, smaller pieces of 2 by 2 cm then extracting the insecticide into a solution using a mixture of solvents. For deltamethrin and alphacypermethrin iso-octan plus 1, 4 dioxan with 0.15% HPLC grade water was used. Dibutyl phthalate was added as the internal standard. For permethrin acetone, 99.9% HPLC grade was used with 99.93% methyl alcohol as the internal standard. After extraction samples were thoroughly shaken to mix and then filtered by water pump suction on 0.45 micrometre membrane filter into a vial. An aliquot of 1 µL of the filtered solution was then injected onto a normal phase isocratic HPLC machine with a *UV *detector. The insecticide quantification was achieved using an internal calibration curve.

### Statistical analysis

Statistical analyses were conducted with SAS, version 9.2. Insecticide residue on the nets after repeated washing was modelled with linear regression. Covariates which were included in this model were the number of washes, drying regimens (shade and spread on ground, sun and spread on ground, shade while hanged and sun while hanged). Mosquito mortalities were calculated using probit regression analysis with machine wash as the reference. Covariates which were included in the model were the number of washes and the washing procedures (washing by hand, machine washing and washing by beating on rocks).

## Results

### Determination of optimal drying regimen

It was observed that, in general, the rate at which LLINs lost insecticide when they were repeatedly washed and dried using four different drying regimens varied with the drying regimen and the net brand. Nets which were air-dried hanging under the shade lost the least amount of insecticide when compared to those that were air-dried using other regimens. For example after 20 washes, the nets washed and air-dried while hanging under the shade significantly (p < 0.0001) retained more insecticide 62.5% compared to those dried hanging in direct sunlight 58.8%, spread on the ground under the shade 57.8% and spread on the ground in direct sunlight 56.3%.

**Figure 1:**
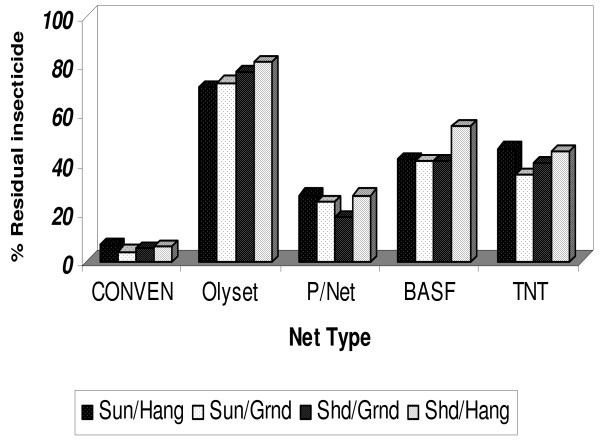
**Percent residual insecticide concentration on each net brand after wash 20 at different drying regimens**. Nets were washed by hand and dried by either hanging in direct sunlight, spread on the ground under the shade, spread on the ground in direct sunlight or hanging on a line under the shade.

**Figure 2:**
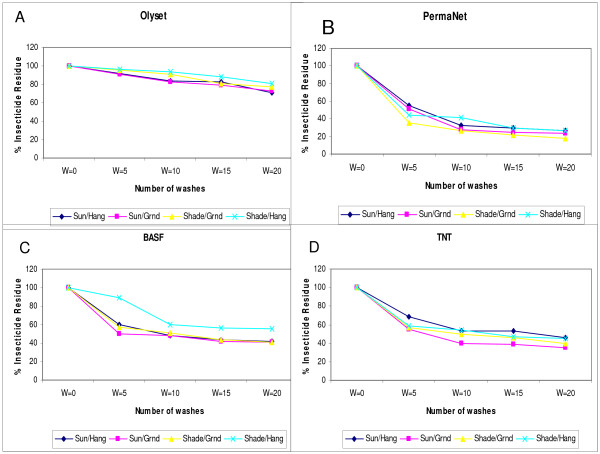
**Percent insecticide residue per net brand and per drying regimens**.

Among the four brands of LLINs used in the current study, Olyset brand of netting retained the highest amount of insecticide > 70% after wash 20. On this net the amount of insecticide retained was highest on the nets that were air-dried hanging under the shade, 80.7% compared to <70.8% that was recorded when dried hanging in direct sunlight, (Figures 1, 2, and Table 1). All the four brands of LLINs evaluated, with exception of TNT, recorded highest amount of insecticide on nets that were dried hanging under the shade. PermaNet recorded 27.5%, BASF 54.5%, and TNT 44%. Overall, after wash 20, PermaNet retained the least amount of insecticide between the four drying regimens 18%-27% compared to BASF and TNT of, 45 - 65% and 42 - 56% respectively; between the four wash drying regimens.

**Table 1:** The percentage residual insecticide per net brand, after repeated washing and drying using four different drying regiments

Net type	Drying regimen	Number of washes				
		W = 0	W = 5	W = 10	W = 15	W = 20
Olyset	Sun/hang	100*	92	84	81.7	70.8
	Sun/Ground	100*	91	83.3	79	73.3
	Shade/Ground	100*	95.1	91	81	77.1
	Shade/Hang	100*	96	94	86	80.7
PerManet	Sun/hang	100*	54.6	31.8	29.6	27.5
	Sun/Ground	100*	51.1	29.5	25.5	23.6
	Shade/Ground	100*	34.5	27.3	21.8	18.2
	Shade/Hang	100*	43.8	41.5	30.2	27.5
BASF	Sun/hang	100*	60	47.9	43.5	42
	Sun/Ground	100*	49.8	47.9	41.8	40.5
	Shade/Ground	100*	57	50.5	44	41.2
	Shade/Hang	100*	80	60	56	54.5
TNT	Sun/hang	100*	69.3	53.9	52.9	46.5
	Sun/Ground	100*	55.1	40	39.1	34.5
	Shade/Ground	100*	56.6	50.8	46.2	40.3
	Shade/Hang	100*	58.5	54	47.4	44.8
Conventional	Sun/hang	100*	43.6	32	16	7.2
	Sun/Ground	100*	28	13.6	7,6	4
	Shade/Ground	100*	34	24.4	11.6	5.2
	Shade/Hang	100*	48	34	12.4	6

Residual insecticide concentration per net brand after repeated washing * The initial insecticide concentrations were as follows: Olyset net, 1000 mg/m², Permanet 55 mg/m², BASF 200 mg/m², TNT 65 mg/m² and Conventional 25 mg/m.

The four drying regimens were also statistically compared on individual net brand. For PermaNet^® ^and TNT, overall there were no significant differences observed between the four drying regimens on these nets (*p *= 0.7944 and 0.4703) respectively). For BASF nets that were dried hanging under the shade performed better compared to nets dried using other regimens p < 0.001. For Olyset, nets that were dried spread on the ground under the shade, performed worse compared to nets that were dried using the other three regimens. All the three drying regimens (sun/ground, sun/hang and shade/hang were statistically significant on Olyset p > 0.0001

The rate of insecticide loss after the above LLINs were repeatedly washed using the three washing procedures were also evaluated. Overall, nets which were washed by machine significantly retained more insecticide after each wash (p < 0.001) compared to nets washed by the two local washing methods. Further analysis on mean values of individual nets, showed that nets washed using WHO protocol, retained more insecticides than when washed by hand rubbing (Table 2).

**Table 2:** Comparison of two washing methods on individual net brands

Variable	Wash type	Mean	95% C.I	SDV	Median	*P *= value
Net type Conventional	Machine	25.7	-4.296-55.7	18.9	24.2	0.183
	Hand	22.3	-5.737-50.34	17.6	18.6	
						
Olyset	Machine	89.7	77.98-101.4	7.35	90.5	0.037
	Hand	84.0	68.23-99.82	9.93	83.8	
						
PermaNet	Machine	40.9	23.97-57.73	10.6	39.5	0.09
	Hand	35.5	22.97-48.03	7.87	35.4	
						
BASF	Machine	64.9	39.02-90.73	16.2	58	0.083
	Hand	57.5	29.46-85.59	17.6	54.2	
						
TNT	Machine	87.3	78.08-96.48	7.41	84.6	0.018
	Hand	60.9	33.02-88.86	22.5	54.0	

Nets were washed using two methods; the standard WHO procedure hereby referred to as machine and hand rubbing with a local detergent OMO.

**Figure 3:**
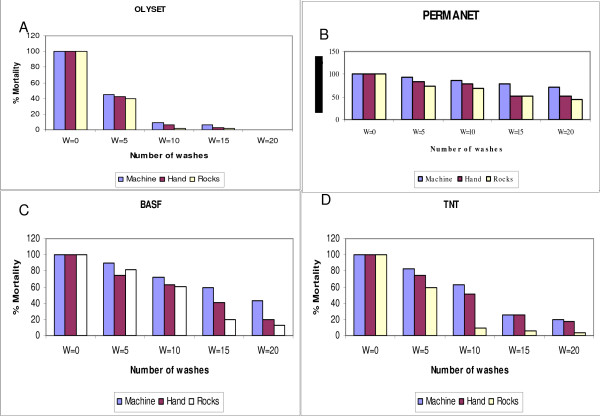
**Mortality rates per net type and per washing procedure**. The figure shows mortality rates of a susceptible strain of *An. gambiae *exposed to four different nets after repeated washing using three different washing procedures.

When the two methods (washing by hand and machine washing) were compared: Olyset retained 89.7% and 84% when washed by machine and hand respectively. Permanet retained 40.9% and 35% when washed by machine and hand respectively. BASF retained 64.9% of the original insecticide and 57.5% when washed by machine and hand respectively, while TNT retained 87.3% compared to 60.9% when washed by machine and hand respectively. These results were statistically significant for Olyest and TNT (p = 0.037) and (0.018) respectively while for PermaNet and BASF the results were not statistically insignificant (0.09 and 0.083 respectively),

Further, the effectiveness of the above LLINs were also evaluated per drying regimen and after repeated washing using three washing procedures. In general, it was observed that the mortalities of *An. gambiae *s.s. recorded 24 hrs after three minutes exposure varied with net type, drying regimen and number of washes (Table 3). For example on PermaNet the mortality reduced from 100 at zero washing to 30% at the 20^th ^washing. For Olyset mortality reduced from 100% to 0%, BASF 100% to 20 and TNT 100 to 15% for the same washing period. Nets washed and air-dried by hanging under the shade showed overall higher percentage mortality than nets which were air-dried hanging or lying on the ground in direct sunlight 100 - 15.8% and 100- 31.3% respectively. The loss in efficacy followed the same trend of insecticide loss. The first five washes were most critical since all the nets except Olyset, exponentially lost up to 50% of their initial efficacy (Figure 3A-D). Between the 5^th ^and 15^th ^washing the loss in efficacy was very gradual while the loss declined from 60 - 40% for nets that were air-dried while hanging under the shade. Similarly, it was observed that, the loss in efficacy corresponded to the reduction in residual insecticide contents for all the nets except for Olyset. Nets that were washed and air-dried while hanging under the shade generally recorded higher percentage mortality, compared to nets which were washed and dried using other regimens. Results of probit analysis modelling for mortalities of mosquitoes showed that there significant differences [p < 0.001]. Nets that were washed and air-dried spread on the ground in direct sunlight were the least effective.

**Table 3:** Mortality rates of *An. gambiae *after exposure on nets repeatedly washed and dried using 4 different drying regimens

Net type/Drying regimen	Number of washes				
Olyset	W = 0	W = 5	W = 10	W = 15	W = 20
Sun/Hang	100	40	0	0	0
Sun/Ground	100	43	5	0	0
Shade/Ground	100	45	5	0	0
Shade/Hang	100	43	0	0	0
PermaNet					
Sun/Hang	100	93	78	45	30
Sun/Ground	100	83	80	43	28
Shade/Ground	100	83	80	65	60
Shade/Hang	100	93	90	85	70
BASF					
Sun/Hang	100	55	43	40	23
Sun/Ground	100	50	35	20	20
Shade/Ground	100	63	55	30	20
Shade/Hang	100	75	65	50	40
TNT					
Sun/Hang	100	45	35	30	20
Sun/Ground	100	53	50	25	15
Shade/Ground	100	58	40	34	28
Shade/Hang	100	53	40	23	15

Nets were washed by hand only. Mortality was measured using the standard WHO recommended 3 minutes cone bioassay test on a susceptible strain of *An. gambiae *ss.

The mean knockdown and mortalities of mosquitoes exposed on nets after repeated washing using two local methods: hand rubbing and beating on rocks are presented in (Table 4). In general, it was observed that nets washed by machine recorded higher knockdown after one hour post exposure and 24-hour mortality respectively compared to nets washed by the other two local washing methods. The knockdown after 20 machine washes was 8%, 68%, 50% and 15% for Olyset, PermaNet, BASF and TNT respectively. The same trend was observed for mortality rates after 24 hrs post exposure. Nets washed by local methods were less effective in killing mosquitoes compared to nets washed by machine. For example, the observed mortalities were: Olyset 1% when nets were washed by beating on rocks compared to 5% when washed by machine, PermaNet recorded 44% compared to 70%, BASF recorded 13% compared to 60 and TNT, 13% compared to 30%. The mortalities for three washing methods was also analysed for each net type with machine wash as the reference using probit regression analysis. Results of modelling for mosquito mortality for Olyset when washed on rocks and machine were statistically insignificant (p > 0.05). However, a significant difference was observed between hand and machine wash p < 0.0001 for the same net type. For PermaNet, hand wash performed better in killing mosquitoes than machine wash (p < 0.0001), while machine performed better than beating on rocks (p < 0.0002). When mortality of mosquitoes was modelled for BASF, machine washing was better than hand washing (p < 0.0001), while for the same net type there was no significant difference between rock and machine (p = 0.0703). Finally, for TNT results of machine and washing by hand were statistically insignificant, but the results of washing same net by rock and machine were significantly different p < 0.0001.

**Table 4:** Knockdown and mortality rates of *An.gambiae *exposed on nets repeatedly washed using WHO procedure and two local washing methods; hand rubbing and beating on rocks

Net type	Machine wash		Hand wash		Washing on Rocks	
Conventional	% Knockdown	% Mortality	% Knockdown	% Mortality	% Knockdown	% Mortality
Unwashed	100	97	100	97	100	97
5X	35	30	40	41	30	28
10X	35	20	35	18	20	15
15X	15	14	10	6	7	4
20X	13	12	4	1	2	0
Olyset						
Unwashed	100	100	100	100	100	100
5X	30	45	45	50	27	34
10X	20	20	23	23	15	8
15X	15	10	11	18	11	2
20X	8	10	6	6	3	1
PermaNet						
Unwashed	100	100	100	100	100	100
5X	95	93	82	94	91	75
10X	91	90	82	87	85	68
15X	85	86	75	79	50	50
20X	68	70	50	72	46	44
BASF						
Unwashed	100	100	100	100	100	100
5X	91	93	86	90	89	82
10X	85	85	86	72	78	61
15X	65	70	46	69	26	20
20X	50	60	38	43	22	13
TNT						
Unwashed	100	100	100	100	100	100
5X	85	86	83	83	55	60
10X	78	80	76	57	38	9
15X	55	61	72	26	21	6
20X	15	30	14	17	7	4

## Discussion

The current study found that the effectiveness and bio-durability of LLINs is influenced by washing methods and drying regimen that are used in the field. Overall, nets that were washed and dried hanging under the shade significantly retained more insecticide and were more effective compared to nets that were washed and dried using other regimens. Generally nets that were dried spread on the ground in direct sunlight lost the most amount of insecticide and were least effective. This finding suggests that contact between wet nets and the ground seem to facilitate transfer of insecticide from netting to the ground or to other surfaces. Unfortunately this is the most preferred method of drying nets in local villages where they are used for malaria vector contro1. The study also found that nets that were washed by the standard WHOPES washing protocol retained more insecticide and were generally more effective in killing mosquitoes than nets that were washed using a local method of beating on rocks. Among the factors that affect efficacy of LLINs, washing seem to be the most important and widely studied. In the current study, it was observed that generally, washing of LLINs gradually removed insecticides on all the four brands of that were evaluated regardless of washing method used. However, the rate at which the insecticide loss occurred was not uniform. It varied by washing method used, (abrasiveness) and the net type (brand). When the bio-efficacy was also measured, using the standard WHO cone bioassay method, mortalities of *An. gambiae *also varied with the number of washes, net brand and washing method. When the residual insecticide contents were compared using statistical procedures, it was also found that, the four drying regimens and the three washing procedures were significantly different and each net brand responded different to each drying and washing method. Nets that were washed by beating on rocks lost insecticide more rapidly and were generally less effective compared to nets washed by hand rubbing and by machine. The finding of the current study suggests that the standard WHOPES washing protocol of using specific machines and soap might be underestimates the actual insecticide loss on nets and overestimating their efficacy and durability under real field conditions where abrasive washing procedures are more abrasive. For example, after the 20^th ^wash, none of the nets achieved >80% mortality on the susceptible strain of *An. gambiae *ss by all the three washing methods.

There are very few studies that have evaluated the effect of different drying regimens on the above LLINs, two of which have been recently recommended as LLINs. The current study attempted to do so. In some unrelated earlier studies, it had been shown that direct sunlight was harmful to pyrethroids based insecticides because UV rays breaks down pyrethrin molecules thereby rendering the insecticide infective. On the basis of this earlier evidence, WHO recommended placing Olyset^® ^in sealed polythene bags and leaving them for a few hours in the sun after washing [1]. The idea was to facilitate insecticide migration to the surface by heating the net at the same time protecting it from uv rays. This recommendation was arbitrary withdrawn because it was cumbersome and the manufacturers of Olyset^® ^claimed that the incorporation of special binders, will, together with high temperatures in the tropics, facilitated automatic insecticide migration to the surface at the same time stabilize them against the effect of UV rays [2]. In the current study it was observed that overall, nets that were dried hanging on line under the shade, generally retained more insecticide. Individually Olyset and BASF brands of LLINs retained more insecticide when dried under the shade. While, the insecticide loss by drying PermaNet and TNT using the four regimens were not statistically different It would have been expected that the trend in insecticide retention by BASF and PermaNet, both of which are polyester based would be the same while that one of TNT and Olyset would also be the same, but this was not observed in the current study. The role played by sunlight in enhancing or rendering the pyrethrins ineffective still remains, controversial. Two studies conducted recently in India, [16,17] showed that exposing Olyset^® ^to sunlight for a few hours enhanced efficacy. The current study also observed that Olyset generally retained more insecticide when it was dried in direct sunlight or hanging under the shade compared to other nets. The high insecticide retention by Olyset^® ^netting recorded in this study and its unavailability on the surface to cause corresponding mosquito mortality suggests that this requirement is still very important. This observation is supported by other studies which have documented that heat accelerates the rate of migration of permethrin molecules in the fibers, thereby increasing the bioavailability of insecticide on the net surface, especially if they are polyethylene based. In one such study Olyset^® ^and PermaNet^® ^were equally exposed to a controlled temperature of 30 and 60°C for 4 hrs after repeated washing, Olyset^®^, regenerated at 60°C to achieve >90% of mosquitoes exposed and not PermaNet^® ^[7]. The current study also observed that even, though PermaNet retained a higher amount of insecticide when dried hanging under the shade compared to other drying regimens, the differences were not statistically significant. The same trend was observed on TNT. In contrast, there were differences in insecticide loss when BSF was air-dried hanging under the shade compared when it was dried hanging in direct sunlight. The findings of the current study, concurs with the results of a similar study conducted in Iran [14]. In the Iran study, Kayedi and others showed that drying PermaNet in direct sunlight for extended period of time of more than 3 hours was harmful. In the current study the four brands of LLINs used were left to dry for a specific period of 4 h.

The current study also compared the bio-efficacy of the above LLINs after repeated washing using WHOPES washing protocol and two local methods of hand rubbing and beating on rocks. The study found that in general nets that were washed using the WHOPES protocol remained effective longer than nets that were washed using local methods. When the differences were modelled by net type and washing method, it was observed that Olyset, PermaNet and BASF performed better when hand washed compared to machine wash. TNT and PermaNet performed better when washed by machine compared to washing on rocks. There was no difference between washing Olyset on rock and machine. The finding of the current study on some of the nets evaluated concurs with the results of a recent study by Sreehari [20]. The study, compared the bio-efficacy of three LLINs: PermaNet^®^, Olyset^® ^and K-O Tab 1, 2, 3^® ^against *An. culicifacies *and *An. stephensi *after repeated washing using machine and hand wash and found that all the three LLINs that were evaluated, retained a significant amount of insecticide after repeated washing by both machine and hand. The study concluded that, nets washed by hand remained effective longer than nets washed by machine. In the current study it was observed that generally, PermaNet^® ^retained its efficacy longer with successive washes using all the three washing methods compared to other three brands of LLINs, but hand washing performed better on this net than machine washing while washing on rocks performed worst. This finding concurs with other studies conducted elsewhere, for example, in Iran Kayedi *et al *[14,21] after evaluating wash resistance of 3 brands after repeated washing found that PermaNet^® ^was more effective. Elsewhere Gimnig *et al *[18] also compared wash resistant and biological activity of five brands of long lasting insecticide treated nets; (PermaNet^®^, Olyset^®^, Dawa^®^, Insector^® ^and Athanor^®^, with a conventionally treated net under laboratory, and found that PermaNet^® ^was the most biologically effective while Olyset^® ^was the most wash-resistant but biologically least biologically effective. The rest of the nets exhibited wide variation in insecticide retention and biological activity.

There are no comparable results for the two newer net brands that were evaluated, (BASF and TNT). But their performances in the current study suggest that they are significantly different in efficacy and durability compared to Olyset and PermaNet. The findings that nets dried spread on the ground generally lost more insecticide and were less effective compared to other drying regimens has major implication on LLINs long-term usage in the rural villages because it is the most preferred drying method. The results suggest that contact between wet net and ground accelerated the rate of insecticide loss from net surfaces. It is possible that insecticide molecules might be migrating from inside the netting to the ground or being denatured by exposure to sunlight. In the current study nets were left in place to dry for a defined period of four hours. In rural areas, it is a normal practice for washed nets to be left outdoor drying for an extended period, sometimes for the whole day. This means that LLINs used in the rural villages might be loosing insecticide at a faster rate depending on the washing frequency and subsequent drying using this regimen.

It is not possible to standardise washing procedures in the field, but there is need to educate rural people on the need to adopt gentle washing on LLINs. The washing of LLINs by beating on hard surfaces such as rocks is a commonly used procedure in the rural areas. This method has not been widely evaluated and there are no comparable studies. The use of this washing procedure is commonly applied on white nettings that have accumulated a lot of dirt. The use of alternative colours for LLINs can indirectly minimise the use of this abrasive method. There is need therefore, to conduct more research and document the effect of this washing method on the long-term use of LLINs. The newer LLINs; BASF^® ^and TNT^®^, whose protective performance was found to be better than Olyset^® ^and lower than PermaNet^® ^have a greater potential of becoming important tools in malaria vector control programmes. These products have not yet undergone extensive evaluation, hence the need for more studies on these products.

## Conclusion

The current study has demonstrated that LLINs that were washed and dried hanging on line under the shade generally, retained more insecticide and remained effective longer than those that were dried using other regimens. The rate of insecticide loss and subsequent reduction in efficacy was also dependant on the washing method used. Nets washed by beating on rocks lost insecticide faster and were least effective compared to nets washed by hand rubbing and washing by machine. The local method of washing LLINs by beating on rocks and air-drying by spreading on the ground in direct sunlight as commonly practiced local villages should be discouraged.

## Competing interests

The authors declare that they have no competing interests.

## Authors' contributions

FKA assisted with data collection, analysis and writing of the manuscript. SOM assisted with critical interpretation of scientific significance of the data, and analysis and review of the manuscript. AVO provided general guidance in scientific methodology for the study and review of manuscript. JMV provided a critical review of the manuscript, and general guidance in data analysis. All authors read and approved the final manuscript.

## References

[B1] WHO Fourth update on long lasting insecticide nets. Current status and programmatic issues http://www.who.int/malaria/publications/atoz/updatellin_4.pdf

[B2] WHO Fifth update on long-lasting insecticidal nets: PermaNet^® ^2.0, a New LLITN Recommended by WHO http://rbm.who.int/docs/updateLLN_5.htm

[B3] WHOPES *Review of Spinosad 0.5% GR and 12% SC, Lambda-Cyhalothrin 10% CS, K-O TAB 1-2-3^®^, Interceptor^® ^2007*. Report of the 10th WHOPES Working Group http://whqlibdoc.who.int/hq/2007/WHO_CDS_NTD_WHOPES_2007_1_eng.pdf

[B4] WHO Recommended long-lasting insecticidal mosquito nets http://www.who.int/whopes/Long-lasting_insecticidal_nets_ok2.pdf

[B5] Gimnig JE Mibangiombe M Chivasse D Mkandala C Mount DL Atieli F Wolkon A Chizani N Hawley WA Campell CH Steketee RW Evaluation of a long lasting mosquito net in Blantyre District, Malawi Am J Trop Med Hyg 2003 3 450 451

[B6] Kayedi G Maxwell MH Kaur C Rehman H Malima R Curtis CF Lines JD Rowland MW Multi-country field trials comparing wash resistance of PermaNet™ and conventional insecticide treated nets against Anopheline and culicine mosquitoes Med Vet Entomol 2005 19 72 83 10.1111/j.0269-283X.2005.00543.x 15752180

[B7] Kulkarni M Update on long lasting insecticidal nets http://www.healthbridge.ca/assets/images/pdf/Malaria/MalariaMatters_15.pdf

[B8] Lindblade KA Dotson E Hawley WA Bayoh N Williamson J Mount D Olang G Vulule J Slutsker L Gimnig J Evaluation of long-lasting insecticidal nets after 2 years of household use Trop Med Int Health 2005 10 1141 1150 10.1111/j.1365-3156.2005.01501.x 16262739

[B9] Smith SC Joshi UB Grabowsky M Selanikio J Nobiya T Aapore T Evaluation of bed-nets after 38 months of household use in Northwest Ghana Am J Trop Med Hyg 2007 77 243 248 18165499

[B10] Killian A Byamukama W Pigeon O Atieli F Duchon S Phan C Long-term field performance of a polyester-based long-lasting insecticidal mosquito net in rural Uganda Malar J 2008 7 49 10.1186/1475-2875-7-49 18355408 PMC2330059

[B11] WHOPES Report of the 12^th ^WHOPES Working Group meeting - Review of Bioflash^® ^GR, Permanet^® ^2.0, Permanet^® ^3.0, Permanet^® ^2.5, Lambda-cyhalothrin LN. 8-11 December 2008, Geneva, World Health Organization, 2008 http://whqlibdoc.who.int/hq/2009/WHO_HTM_NTD_WHOPES_2009_1_eng.pdf

[B12] WHOPES Report of the 13th WHOPES Working Group meeting - Review of Olyset^® ^LN, Dawaplus^® ^2.0 LN, Tianjin Yorkool^® ^LN. 28-30 July 2009, Geneva, World Health Organization, 2009 http://whqlibdoc.who.int/publications/2009/9789241598712_eng.pdf

[B13] WHOPES Guidelines for Laboratory and field testing of Long Lasting Insecticidal Mosquito Nets. 2005 http://whqlibdoc.who.int/hq/2005/WHO_CDS_WHOPES_GCDPP_2005.11.pdf

[B14] Kayedi MH Lines JD Haghdoosta AA Vatandoostd MH Rassid Y Khamisabadi K Evaluation of the effects of repeated hand washing, sunlight, smoke and dirt on the persistence of deltamethrin on insecticide-treated nets Trans R Soc Trop Med Hyg 2008 102 811 816 10.1016/j.trstmh.2008.05.025 18579169

[B15] Morris SE Davies NW Brown PH Groom T Effect of drying conditions on pyrethrins content Industrial Crops and Products 2006 23 9 14 10.1016/j.indcrop.2005.01.007

[B16] Jeyalakshmi T Shanmugasundaram R Balakrishna MP Comparative efficacy and persistency of permethrin in Olyset net and conventionally treated nets against *and Anopheles stephensi* J Am Mosq Cont Assoc 2006 22 107 110 10.2987/8756-971X(2006)22[107:CEAPOP]2.0.CO;2 16646331

[B17] Sharma SK Upadhyay AK Haque MA Padhan K Tyagi PK Ansari MA Dash AP Wash resistance and bioefficacy of Olyset net -- a long-lasting insecticide-treated mosquito net against malaria vectors and nontarget household pests J Med Entom 2006 43 884 888 10.1603/0022-2585(2006)43[884:WRABOO]2.0.CO;2 17017224

[B18] Gimnig JE Lindblade KL Mount DL Atieli FK Crawford S Wolkon A Hawley WA Dotson EM Laboratory wash resistance of long-lasting insecticidal nets Trop Med Int Health 2005 10 1022 1029 10.1111/j.1365-3156.2005.01481.x 16185237

[B19] Armstrong DW Young X Han SM Menges RA Direct liquid chromatographic separation of racemates with an a-cyclodextrin bonded phase Anal Chem 1987 59 2584 2596 10.1021/ac00148a014 3688447

[B20] Sreehari1 U Raghavendra1 K Rizvi MMA Dash1 AP Wash resistance and efficacy of three long-lasting insecticidal nets assessed from bioassays on *Anopheles culicifacies *and *Anopheles stephensi* Trop Med Int Health 2009 14 597 602 10.1111/j.1365-3156.2009.02252.x 19228347

[B21] Kayedi MH Lines JD Haghdoosta AA Behrahi A Khamisabadi K Entomological evaluation of three brands of manufactured insecticidal nets and of conventionally treated with deltamethrin, after repeated washing Ann Trop Med Parasitol 2007 101 449 456 10.1179/136485907X176481 17550651

